# Diversity of *Streptomyces* spp. from mangrove forest of Sarawak (Malaysia) and screening of their antioxidant and cytotoxic activities

**DOI:** 10.1038/s41598-019-51622-x

**Published:** 2019-12-03

**Authors:** Jodi Woan-Fei Law, Kok-Gan Chan, Ya-Wen He, Tahir Mehmood Khan, Nurul-Syakima Ab Mutalib, Bey-Hing Goh, Learn-Han Lee

**Affiliations:** 1 Institute of Biomedical and Pharmaceutical Sciences, Guangdong University of Technology, Guangzhou, 510006 P.R. China; 2 Novel Bacteria and Drug Discovery (NBDD) Research Group, Microbiome and Bioresource Research Strength, Jeffrey Cheah School of Medicine and Health Sciences, Monash University Malaysia, 47500 Bandar Sunway, Selangor Darul Ehsan Malaysia; 3 Division of Genetics and Molecular Biology, Institute of Biological Sciences, Faculty of Science, University of Malaya, 50603 Kuala Lumpur, Malaysia; 4 International Genome Centre, Jiangsu University, Zhenjiang, China; 5 Microbial Quorum Sensing and Synthetic Biology, School of Life Sciences and Biotechnology, Shanghai Jiao Tong University, Dongchuan Road, #800, Shanghai, 200240 China; 6 Biofunctional Molecule Exploratory Research Group (BMEX), School of Pharmacy, Monash University Malaysia, 47500 Bandar Sunway, Selangor Darul Ehsan Malaysia; 7 Institute of Pharmaceutical Sciences (IPS), University of Veterinary & Animal Sciences (UVAS) Out Fall Road, Lahore, Pakistan; 8 UKM Medical Molecular Biology Institute (UMBI), UKM Medical Centre, Universiti Kebangsaan Malaysia, Kuala Lumpur, Malaysia; 9 Health and Well-being Cluster, Global Asia in the 21st Century (GA21) Platform, Monash University Malaysia, 47500 Bandar Sunway, Malaysia

**Keywords:** Bacteriology, Marine microbiology

## Abstract

Streptomycetes have been the center of attraction within scientific community owing to their capability to produce various bioactive compounds, for instance, with different antimicrobial, anticancer, and antioxidant properties. The search for novel *Streptomyces* spp. from underexplored area such as mangrove environment has been gaining attention since these microorganisms could produce pharmaceutically important metabolites. The aim of this study is to discover the diversity of *Streptomyces* spp. from mangrove in Sarawak and their bioactive potentials — in relation to antioxidant and cytotoxic activities. A total of 88 *Streptomyces* isolates were successfully recovered from the mangrove soil in Kuching, state of Sarawak, Malaysia. Phylogenetic analysis of all the isolates and their closely related type strains using 16S rRNA gene sequences resulted in 7 major clades in the phylogenetic tree reconstructed based on neighbour-joining algorithm. Of the 88 isolates, 18 isolates could be considered as potentially novel species according to the 16S rRNA gene sequence and phylogenetic analyses. Preliminary bioactivity screening conducted on the potential novel *Streptomyces* isolates revealed significant antioxidant activity and notable cytotoxic effect against tested colon cancer cell lines (HCT-116, HT-29, Caco-2, and SW480), with greater cytotoxicity towards SW480 and HT-29 cells. This study highlighted that the Sarawak mangrove environment is a rich reservoir containing streptomycetes that could produce novel secondary metabolites with antioxidant and cytotoxic activities.

## Introduction

The remarkable contribution of microbes towards the area of drug discovery has ultimately improved human welfare around the world^1^. This is due to their capability in producing various useful natural products which later became the source of countless active ingredients of medicines^2–4^. In this regard, bacteria belonging to the genus *Streptomyces* have been acknowledged as the producers of many bioactive compounds, which makes them to be important microorganisms for drug discovery^5,6^. *Streptomyces* is documented as the major genus of the order *Streptomycetales* within the class *Actinobacteria*^7–10^.

Streptomycetes are complex filamentous Gram-positive bacteria with morphology resembles those of fungi^11–13^. The complexity of streptomycetes can be observed through their complicated developmental life cycle and their large genome size of more than 8 Mbp with high G + C content,often associated with their ability to prosper and survive in different environments^13–16^. Studies also revealed the presence of over 20 biosynthetic gene clusters related to biosynthesis of secondary metabolites in the large genome of streptomycetes. This may account for the production of structurally diverse bioactive secondary metabolites^10,13,17^. Due to the production of various useful compounds from streptomycetes including enzymes, pigments, and compounds possessing antimicrobial, anticancer, antioxidant, immunosuppressive and other important bioactivities^17–21^, these bacteria have been greatly explored for wide-range of applications. Currently, there are approximately 843 *Streptomyces* species isolated from different environments (www.bacterio.net)^22^.

Recently, researchers expressed interest in seeking novel streptomycetes from underexplored areas to increase the probabilities of discovering new compounds or therapeutic agents^23^. Mangrove environments are often underexplored but contains good resources for the isolation of novel streptomycetes^24^. It is known that the mangrove environments are constantly experiencing environmental variations such as changes in tidal gradient and salinity^19,25^. Despite these dynamic environmental factors, mangrove forests have always been home to various plants and animals. Instead, these unusual environmental changes may be the driving force for the development of microbial species diversity and adaptation of metabolic pathways that could be responsible to generate certain unique properties of microorganisms^19,26^. In this context, the study of streptomycetes from mangrove may provide a better prospect of uncovering novel *Streptomyces* spp. which may subsequently bring about the discovery of valuable bioactive molecules^27,28^.

Researchers are still actively studying the diversity of the microbial community in the phylum *Actinobacteria* originating from different environments and countries, often due to their ecological importance and biotechnological benefits^29–31^. However, there are limited number of studies reported on the diversity of streptomycetes especially in Malaysia and thus the population of this bacteria in Malaysia environments are poorly understood. Recent studies provide an increasing evidence on novel *Streptomyces* spp. isolated from Malaysia mangrove forests that could be valuable resource for antioxidant and anticancer compounds. For examples, crude extracts of *Streptomyces antioxidans* sp. nov. MUSC 164^T^ and *Streptomyces mangrovisoli* sp. nov. MUSC 149^T^ exhibited strong antioxidant activity^23,32^. *Streptomyces malaysiense* sp. nov. MUSC 136^T^ discovered by Ser *et al*.^33^ was found to possess strong antioxidant activity and exhibit high cytotoxicity against colon cancer cell line HCT-116. Malaysia is categorized as a mangrove-rich country in Asia and many mangrove areas in Sarawak are mostly in pristine state^34,35^. Therefore, this creates an important opportunity to explore the streptomycetes present in Sarawak mangrove forest together with their antioxidant and cytotoxic potentials.

Natural compounds have played an essential role in preventing or treating cancer, which is a major public health concern^36–38^. It is known that the development of cancer is linked to oxidative stress - a condition recognized by the imbalance between production of reactive oxygen species (ROS) and the competence to counteract the damage caused by ROS through antioxidants^39,40^. There is no doubt that researchers have been continuously looking for effective natural antioxidant and anticancer agents from natural sources including microorganisms^41^. For instance, a recent study conducted by Rao *et al*.^42^ reported the discovery of three pure compounds possessing antioxidant activity from a mangrove-derived *Streptomyces coelicoflavus* BC 01, namely 5-amino-2-(6-(2-hydroxyethyl)-3-oxononyl) cyclohex-2-enone **(BC 01_C1)**, 8-(aminomethyl)-7-hydroxy-1-(1-hydroxy-4-(hydroxylmethoxy)-2,3-dimethylbutyl)-2-methyl dodecahydro phenanthren-9(1H)-one **(BC 01_C2)**, and 1-((E)-2-ethylhex-1-en-1-yl)2-((E)-2-ethylidenehexyl)cyclohexane-1,2-dicarboxylate **(BC 01_C3)**. Apart from that, two novel bioactive compounds known as neoantimycins A and B were discovered from mangrove-derived *Streptomyces antibioticus* H12-15^43^. The compounds showed cytotoxicity against human breast adenocarcinoma (MCF-7) cell line (IC_50_ > 50 µg/mL by both compounds), human glioblastoma (SF-268) cell line (IC_50_ of 33.6 µg/mL by neoantimycin A; IC_50_ of 41.6 µg/mL by neoantimycin B), and human lung cancer (NCl-H460) cell line (IC_50_ > 50 µg/mL by both compounds)^43^. Mangamuri *et al*.^44^ found a compound known as 2-methyl butyl propyl phthalate produced by mangrove-derived *Streptomyces cheonanensis* VUK-A, which exerted significant cytotoxic effect on human breast adenocarcinoma (MDA-MB-231), human cervical cancer (HeLa), human ovarian cyst adenocarcinoma (OAW42), and MCF-7 cell lines. In fact, the genus *Streptomyces* is a good source of chemotherapeutic agents verified through the discovery of several clinically important anticancer medicines such as mitomycin C^45^, dactinomycin^46^, doxorubicin (synonym adriamycin)^47^, and bleomycin^13,48,49^.

This study aims to explore the diversity of *Streptomyces* spp. from mangrove in Sarawak and screen them to determine potential sources for antioxidant and cytotoxic secondary metabolites. Sarawak mangrove forest mostly remains undisturbed, thus it is foreseen that this location could provide a rich supply of actinobacteria. To the best of our knowledge, this is the first report on the diversity and bioactive properties of streptomycetes from mangrove environments in Sarawak.

## Results

### Isolation of *Streptomyces* spp

Isolation of actinomycetes from environmental samples usually involve pre-treatments and the use of antibiotics as selective agents on culture media plates^50,51^. The study involved wet heat pre-treatment of sediments prior to isolation of streptomycetes. This is to reduce the number of undesirable bacteria present in the sediments that could often overrun media plates^52,53^. The isolation media were supplemented with cycloheximide to suppress the growth of fungi and nalidixic acid to suppress the growth of Gram-negative bacteria^54,55^.

A series of media plates were used for the isolation and characterization of streptomycetes in the present study. According to the typical cultural characteristics of a streptomycete (e.g. filamentous colonies with the development of dry, powdery-cottony aerial mycelium and non-fragmenting substrate mycelium) on media plate^12,56,57^, a total of 88 putative *Streptomyces* isolates were successfully isolated from 8 types of isolation media: ISP 2 (*n* = 3), ISP 5 (*n* = 8), ISP 6 (*n* = 24), ISP 7 (*n* = 9), SCA (*n* = 3), AIA (*n* = 5), NA (*n* = 22), and LB (*n* = 14). Based on the number of isolates recovered from each medium, it is noticeable that ISP 6 (peptone yeast extract 6 iron agar) was the most suitable medium for the isolation of *Streptomyces* in this study. This result is in agreement with others, which also recorded good growth of streptomycetes on ISP 6^58–60^. Mangrove soil samples were collected from 7 sites and labelled as KTTAS 1, KTTAS 2, KTTAS 3, KTTAS 4, KTTAS 5, KTTAS 6, and KTTAS 7 which contributes to 20, 21, 25, 4, 2, 1, and 15 isolates respectively, of the total number of isolates.

### Diversity of *Streptomyces* isolates

**Figure 1:**
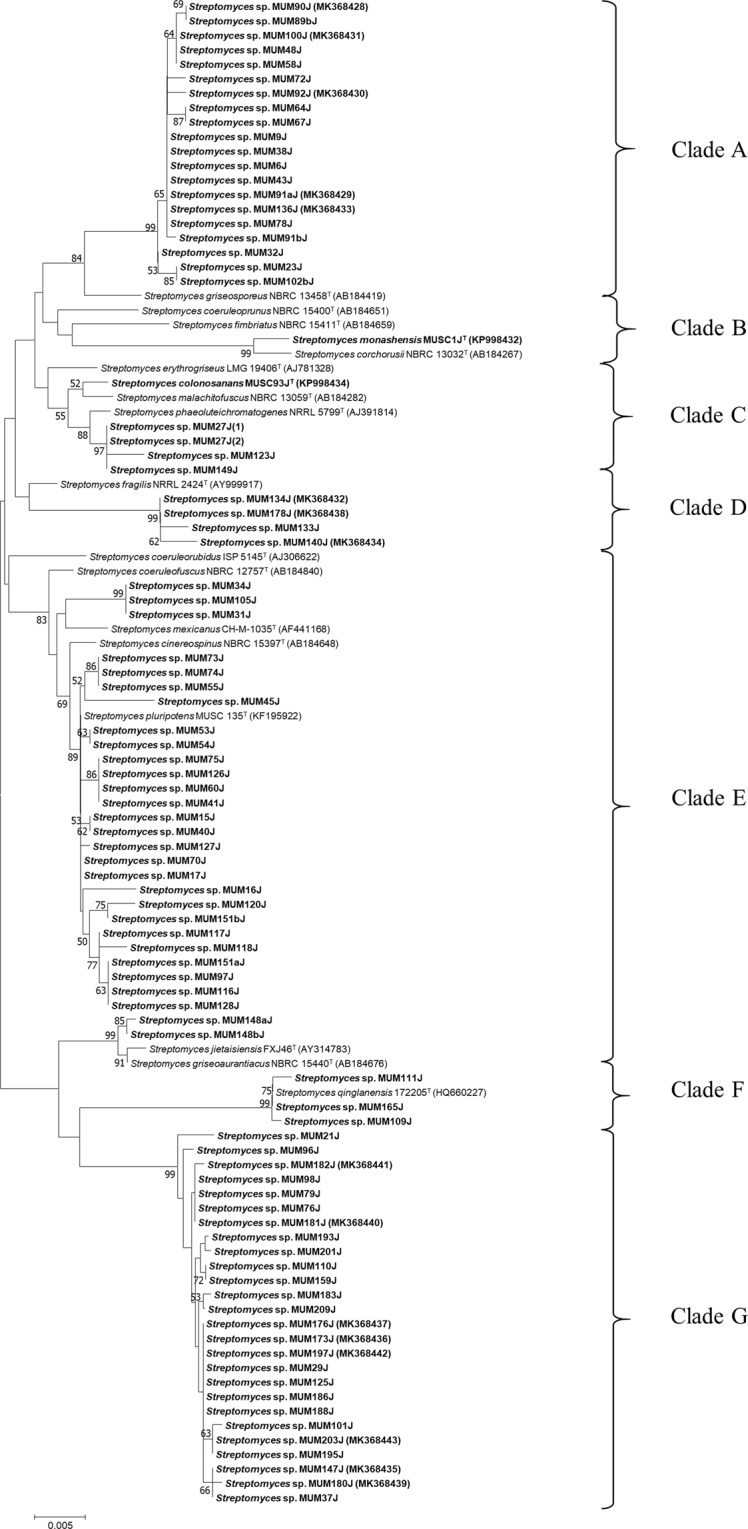
Neighbour-joining phylogenetic tree based on 16S rRNA gene sequences showing the relationship between the 88 *Streptomyces* sp. isolates and their closely related type strains. Bootstrap value based on 1000 resampled datasets are shown at branch nodes. Bar, 0.005 substitutions per site.

These isolates were further identified and confirmed as *Streptomyces* sp. based on molecular analysis of 16S rRNA gene sequences. The 16S rRNA gene sequences of these isolates were compared with related type strains retrieved from DDBJ/EMBL/GenBank, and the results displayed percentages of pairwise sequence similarity ranged from 98.03% to 100%. Their 16S rRNA gene sequences were utilized for the reconstruction of neighbor-joining phylogenetic tree consisting 88 isolates of *Streptomyces* together with their closely related type strains to understand their taxonomic relationships (Fig. 1). Among these isolates, MUSC 1J^T^ and MUSC 93J^T^ have been confirmed as novel species. These two novel species, with the designated names *Streptomyces monashensis* sp. nov. MUSC 1J^T^ and *Streptomyces colonosanans* sp. nov. MUSC 93J^T^, were reported separately in earlier studies^25,26^.

Neighbour-joining phylogenetic tree constructed based on 16S rRNA gene sequences revealed 7 major clades, designated as Clade A-G; with 20 *Streptomyces* isolates were assigned to Clade A, 1 isolate was assigned to Clade B, 5 isolates assigned to Clade C, 4 isolates were assigned to Clade D, 29 isolates were assigned to Clade E, 3 isolates were assigned to Clade F, and 26 isolates were assigned to Clade G (Fig. 1).

The phylogenetic analysis demonstrated correlation between several isolates with type strains that possessed important bioactivities. In Clade B, *S*. *monashensis* MUSC 1J^T^ showed the highest percentage of 16S rRNA gene sequence similarity of 98.70% to *Streptomyces corchorusii* NBRC 13032^T^. The phylogenetic analysis showed that *S*. *monashensis* MUSC 1J^T^ formed a monophyletic clade with type strain *S*. *corchorusii* NBRC 13032^T^ at 99% bootstrap value (Fig. 1). Apparently, *S*. *corchorusii* AUBN_1_/7, a strain isolated from marine sediment is capable of producing cytotoxic compounds such as resistomycin and tetracenomycin D^61^. Both pure compounds showed strong cytotoxicity against gastric adenocarcinoma (HMO2) and human liver cancer (HepG2) cell lines. The LC_50_ values of resistomycin against HMO2 cells was 0.012 µg/mL and HepG2 cells was 0.015 µg/mL, whilst the LC_50_ values of tetracenomycin D against HMO2 cells was 0.016 µg/mL and HepG2 cells was 0.021 µg/mL.

In Clade C, *S*. *colonosanans* MUSC 93J^T^ displayed the highest 16S rRNA gene sequence similarity of 99.24% to type strain *Streptomyces malachitofuscus* NBRC 13059^T^, forming a monophyletic clade at 52% bootstrap value (Fig. 1). Sajid *et al*.^62^ isolated *Streptomyces* sp. CTF9 with promising antifungal activity from soil samples of saline agricultural farmlands in Pakistan. This isolate was identified as *S*. *malachitofuscus* and it produced two active antifungal metabolites identified as phenylacetic acid and indolyl-1-lactic acid.

In addition, *Streptomyces* sp. MUM 133J, MUM 134J, MUM 140J, and MUM 178J exhibited highest percentage of 16S rRNA gene sequence similarity (98.06–98.65%) to type strain *Streptomyces fragilis* NRRL 2424^T^, forming a distinct phylogenetic clade as illustrated in Clade D (Fig. 1). *S*. *fragilis* is known as the producer of azaserine, which is a tumor-inhibiting antibiotic^63,64^.

For Clade E, 24 out of 29 *Streptomyces* isolates are closely related to type strain *Streptomyces pluripotens* MUSC 135^T^, with highest percentage of 16S rRNA gene sequence similarities ranged from 99.25% to 100%. *S*. *pluripotens* MUSC 135^T^ was first discovered by Lee *et al*.^65^ from mangrove soil collected at Tanjung Lumpur, Malaysia. The research group also reported the production of broad-spectrum bacteriocin from *S*. *pluripotens* that successfully inhibited methicillin-resistant *Staphylococcus aureus*. Furthermore, crude extract of *S*. *pluripotens* was also found to exhibit antioxidant and cytotoxic activities^66,67^. Two *Streptomyces* isolates, MUM 148aJ and MUM 148bJ exhibited highest percentage of 16S rRNA gene sequence similarity to type strain *Streptomyces griseoaurantiacus* NBRC 15440^T^ with 99.62% and 99.85% identities respectively, and they formed a distinct phylogenetic clade with *S*. *griseoaurantiacus* NBRC 15440^T^ and *Streptomyces jietaisiensis* FXJ46^T^ at 99% bootstrap value, indicating high stability of the grouping (Fig. 1). Prashanthi *et al*.^68^ reported the isolation of *S*. *griseoaurantiacus* from soil samples that produced a yellow pigment which demonstrated *in vitro* anticancer activity against HeLa and HepG2 cell lines. The pigment exhibited strong cytotoxic effect towards HeLa cells with IC_50_ value of 1.8 µg/mL and HepG2 cells with IC_50_ value of 1.41 µg/mL after 72 hours treatment using MTT cell viability assay.

*Streptomyces* sp. MUM 109 J, MUM 111 J, and MUM 165 J exhibited highest percentage of 16S rRNA gene sequence similarity (99.85–100%) to type strain *Streptomyces qinglanensis* 172205^T^ and the phylogenetic analysis further supports that they are closely related to each other as observed in Clade F (Fig. 1). *S*. *qinglanensis* was first discovered and isolated from mangrove sediment at China by Hu *et al*.^69^.

As for Clade G, all *Streptomyces* isolates except *Streptomyces* sp. MUM 176J exhibited highest percentage of 16S rRNA gene sequence similarity (98.20–98.86%) to type strain *Streptomyces coeruleorubidus* ISP 5145^T^. *Streptomyces* sp. MUM 176 J showed highest percentage of 16S rRNA gene sequence similarity of 98.25% to type strain *Streptomyces coeruleoprunus* NBRC 15400^T^. The phylogenetic analysis showed that all 26 *Streptomyces* isolates formed a distinct phylogenetic clade with high bootstrap value of 99% (Fig. 1). However, these isolates appeared to be in a clade that was distinct from the type strains *S*. *coeruleorubidus* ISP 5145^T^ and *S*. *coeruleoprunus* NBRC 15400^T^. *S*. *coeruleorubidus* obtained from Egyptian soil was previously reported to produce antitumor/antibiotic activity by El-Sayed *et al*.^70^. The crude extract of this isolate was active in decreasing the survival of HepG2 cells and HCT-116 cells with IC_50_ values of 19.0 µg/mL and 26.8 µg/mL respectively.

Phylogenetic analysis based on the 16S rRNA gene sequences illustrated the relationship between these *Streptomyces* isolates. Taxonomic studies suggested that some isolates have the potential to be assigned as novel species. Besides, it can be deduced that the isolates in this study may produce interesting bioactive secondary metabolites since several of their closely related type strains exhibited bioactivities such as antioxidant, anticancer, and antimicrobial. Therefore, this led to the search of novel *Streptomyces* spp. and the exploration of their potential bioactivities.

### Diversity and phylogeny of potentially novel *Streptomyces* isolates

**Figure 2:**
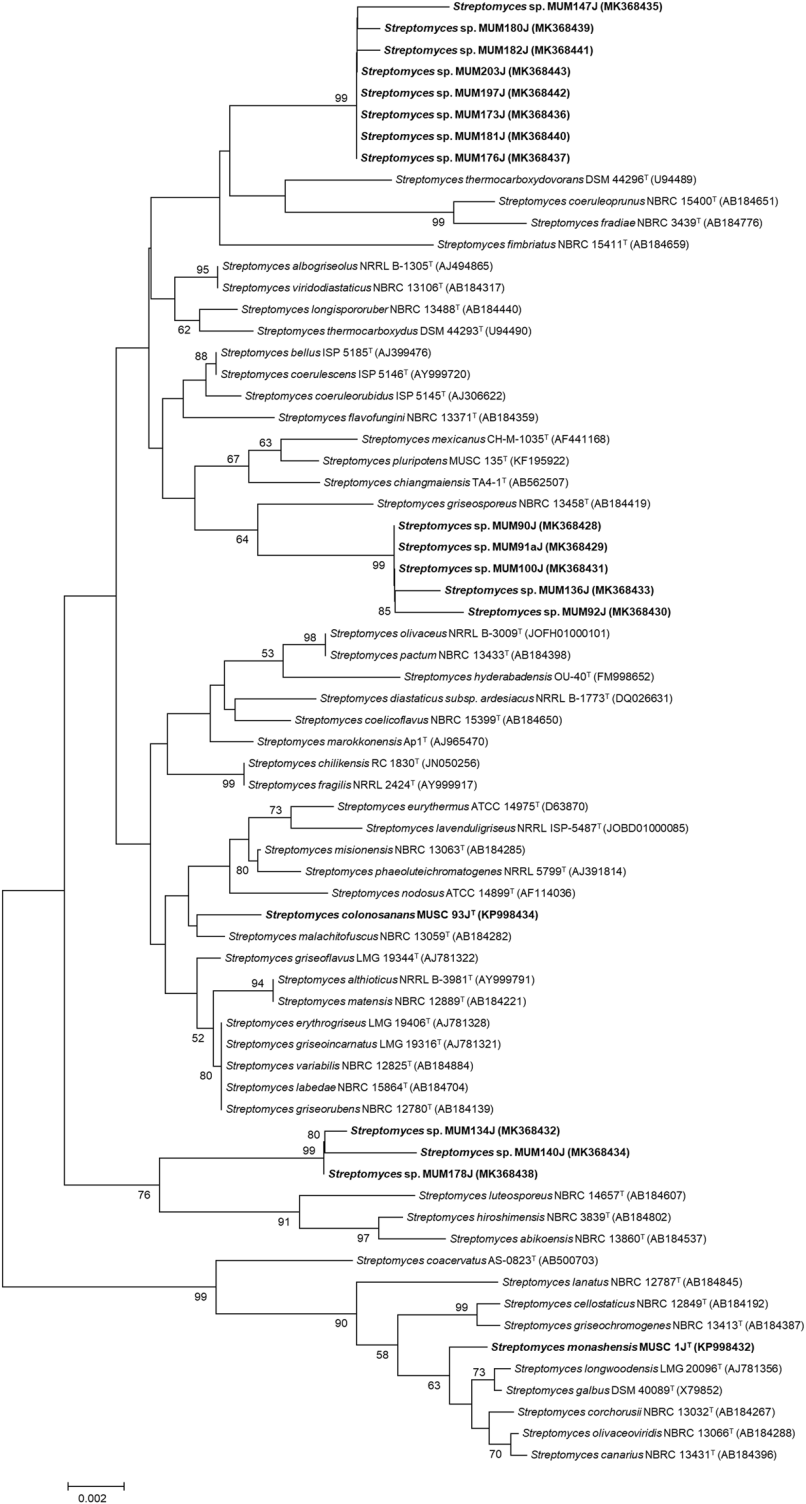
Neighbour-joining phylogenetic tree based on 16S rRNA gene sequences showing relationship between 18 potentially novel *Streptomyces* sp. and their closely related type strains. Bootstrap value based on 1000 resampled datasets are shown at branch nodes. Bar, 0.002 substitutions per site.

Detailed phylogenetic analysis and pairwise comparison of 16S rRNA gene sequences of each *Streptomyces* isolate with its related type strains were conducted, and the results suggested that 18 out of the 88 *Streptomyces* isolates demonstrated high possibilities of novel species discovery. This taxonomic investigation of streptomycetes was in accordance with the approach performed by Lee *et al*.^28^. The phylogenetic relationship between the 18 potentially novel *Streptomyces* isolates and their closest related type strains is shown in Fig. 2.

Notably, several type strains of *Streptomyces* listed in the neighbour-joining phylogenetic tree (Fig. 2) were first discovered as novel species originated from soil samples. For instances, *Streptomyces thermocarboxydovorans* DSM 44296^T^ and *Streptomyces thermocarboxydus* DSM 44293^T^ isolated from soil were first described and reported by Kim *et al*.^71^; *Streptomyces hyderabadensis* OU-40^T^ was first discovered by Reddy *et al*.^72^ from farm soil in Southern India; *Streptomyces mexicanus* CH-M-1035^T^ was first discovered by Petrosyan *et al*.^73^ from soil in Mexico; and *Streptomyces pluripotens* MUSC 135^T^ was first isolated from mangrove soil in Malaysia and reported by Lee *et al*.^65^. Hence, it can be anticipated to unveil novel species from soils of the unexplored Sarawak mangrove forest.

Moreover, some of the closest related type strains were reported to exhibit bioactivities relating to antioxidant and antitumor/anticancer. For example, the crude methanolic extract of *Streptomyces pluripotens* exhibited significant antioxidative activity and was found to be cytotoxic against several human cancer cell lines such as colon cancer cell lines (HCT-116, HT-29, Caco-2, SW480), lung carcinoma cell line (A549), breast adenocarcinoma cell line (MCF-7), prostate cancer cell line (DU145), and cervical cancer cell line (Ca Ski)^66^. Balachandran *et al*.^74^ reported that *Streptomyces galbus* showed cytotoxicity towards A549 cell line and the active compound was found to be 2,3-dihydroxy-9,10-anthraquinone. *Streptomyces griseorubens* was previously reported to showed antitumor activity against HeLa cell line, human oral epidermoid cancer (KB) cell line and human hepatocarcinoma (SMMC7721) cell line^75^. Also, Reda (2015)^76^ reported the production of L-glutaminase by *Streptomyces canarius* which had anticancer activity against HepG2, HeLa, and HCT-116 cells. These findings generally infer that the potentially novel *Streptomyces* isolates could be able to produce biologically active secondary metabolites such as antioxidant or anticancer agents.

### Antioxidant activity of extracts from potentially novel *Streptomyces* isolates

**Figure 3:**
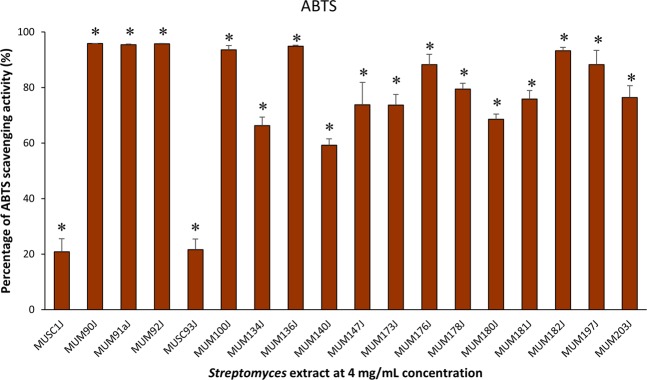
ABTS radical scavenging activity of extracts from potentially novel *Streptomyces* sp. isolates. Symbol (*) indicates p < 0.05 significant difference between the extract and control (without extract).

**Figure 4:**
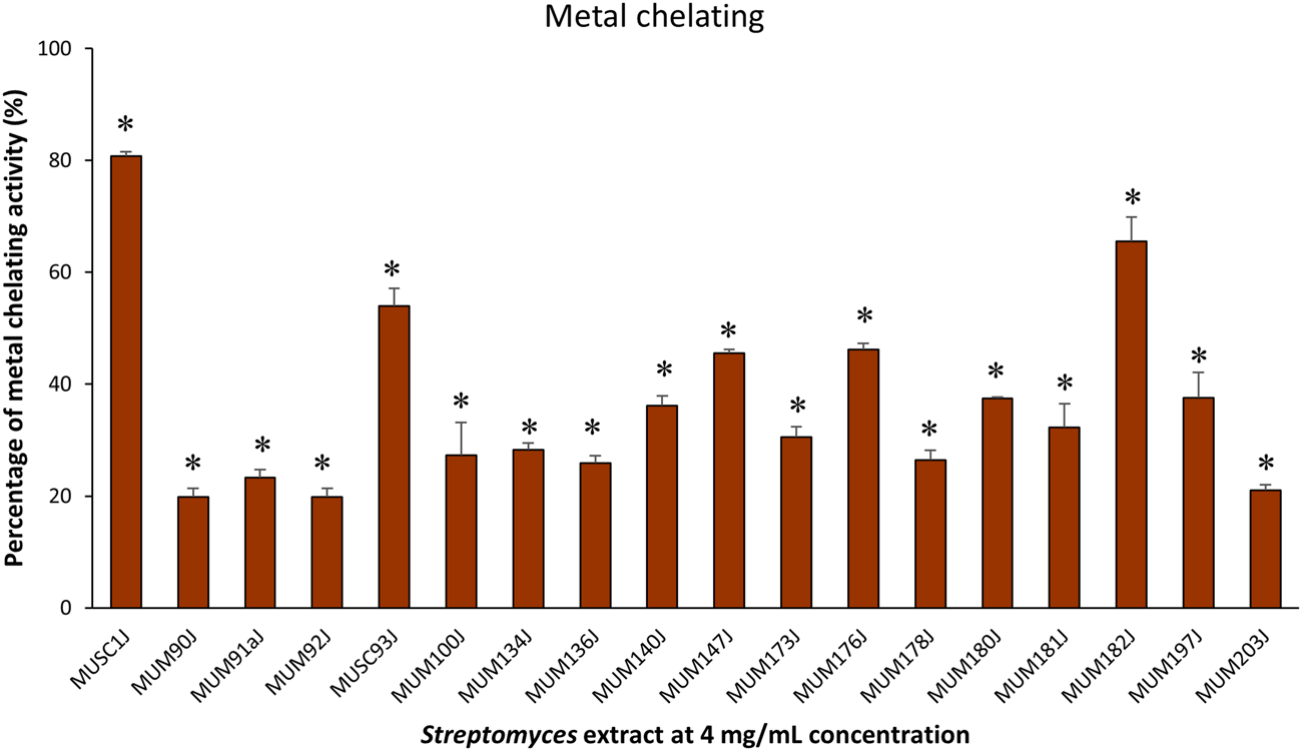
Metal chelating activity of extracts from potentially novel *Streptomyces* sp. isolates. Symbol (*) indicates p < 0.05 significant difference between the extract and control (without extract).

**Figure 5:**
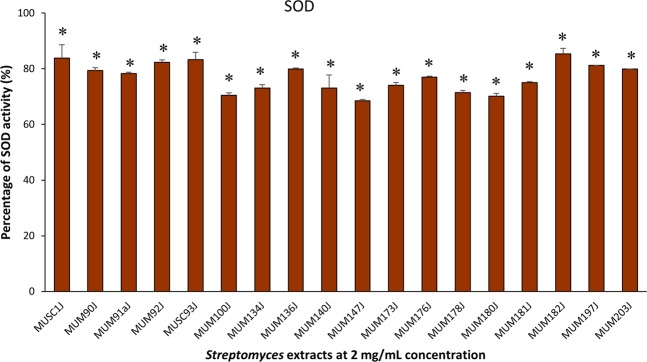
SOD-like activity of extracts from potentially novel *Streptomyces* sp. isolates. Symbol (*) indicates p < 0.05 significant difference between the extract and control (without extract).

Preliminary screening of antioxidant activity of the 18 potentially novel isolates was conducted using a high-throughput screening model involving the use of 96-well plates. The outcomes of preliminary screening using ABTS, metal chelating, and SOD assays are presented in Figs 3–5 respectively. Overall, all tested extracts exerted statistically significant antioxidant activity via ABTS, metal chelating, and SOD assays. It can be observed that the extracts were capable of scavenging ABTS free radicals ranged from 20.87 ± 4.69% to 95.82 ± 0.05% at 4 mg/mL concentration (Fig. 3). For the metal chelating activity, the extracts exhibited significant activity ranged from 19.84 ± 1.53% to 80.77 ± 0.84% at 4 mg/mL concentration (Fig. 4). In this assay, antioxidative potential of the extracts was emphasized by prohibiting transition metals from promoting the generation of ROS^33^. Besides, the *Streptomyces* extracts demonstrated strong SOD-like activity ranged from 68.54 ± 0.39% to 85.35 ± 1.93% at the highest tested concentration of 2 mg/mL (Fig. 5). Most of the extracts showed high activity (>50%) in ABTS and SOD assays. However, only 3 extracts (MUSC 1J^T^, MUSC 93J^T^, and MUM 182J) showed more than 50% of metal chelating activity. Among the tested extracts, only MUM182J exhibited more than 50% activity in all antioxidant assays. These results may suggest that most extracts are effective in scavenging ABTS free radicals and preventing generation of superoxide anion radicals but not as good at chelating metal ions.

### Total phenolic content (TPC) of methanolic extracts and antioxidant activity

**Figure 6:**
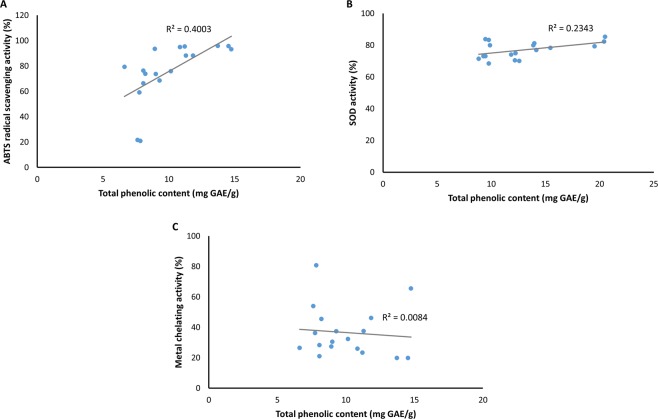
Correlation between total phenolic content and antioxidant capacity of 18 *Streptomyces* methanolic extracts. The relationship was observed in three different antioxidant assays: (**A**) ABTS, (**B**) SOD, and (**C**) metal chelating.

The TPC of extracts from the 18 potentially novel streptomycetes was evaluated. This analysis attempted to correlate the TPC and antioxidant activity across different *Streptomyces* methanolic extracts. The correlation coefficient (*R*^2^) was estimated to determine the relationship between the TPC and the antioxidant capacity of the 18 methanolic extracts (Fig. 6). The results revealed that the correlation coefficient between TPC and antioxidant capacity as analyzed by three different assays were very small; where the highest was observed in ABTS radical scavenging activity (*R*^2^ = 0.4003) (Fig. 6A), followed by SOD activity (*R*^2^ = 0.2343) (Fig. 6B), and metal chelating activity (*R*^2^ = 0.0084) (Fig. 6C).

### Cytotoxic activity of extracts from potentially novel *Streptomyces* isolates

**Figure 7:**
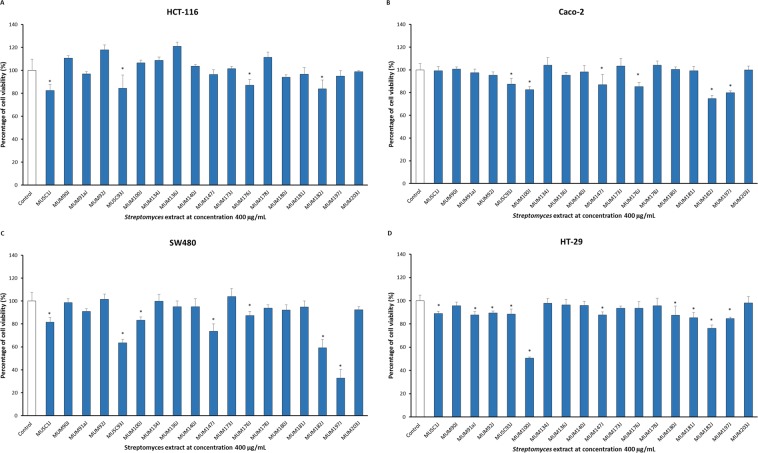
Cytotoxic activity of extracts from potentially novel *Streptomyces* isolates against colon cancer cell lines. The measurement of cell viability was done using MTT assay and the concentration of extract was 400 µg/mL. The graphs show cytotoxicity effect of the extracts against: (**A**) HCT-116, (**B**) Caco-2, (**C**) SW480, and (**D**) HT-29. All data are expressed as mean ± standard deviation and significance level are set as 0.05. Symbol (*) indicates p < 0.05 significant difference between the cells treated with extract and control (without extract).

The present study examined the cytotoxic potential of extracts in four high-throughput models against human colon cancer cell lines: HCT-116, HT-29, Caco-2, and SW480, with extracts examined at 400 µg/mL. The results illustrated that the extracts exhibited varying cytotoxicity against different colon cancer cell lines (Fig. 7). Of all the tested human colon cancer cell lines, the cytotoxic effect of the extracts was least notable against HCT-116 cell line, as only a total of 4 out of 18 *Streptomyces* extracts tested (MUSC 1J, MUSC 93J, MUM 176J, and MUM 182 J) exhibited significant cytotoxicity towards HCT-116 cells, with cell viability ranged from 82.3 ± 5.3% to 86.9 ± 5.0% (Fig. 7A). As for the Caco-2 cell line, 6 *Streptomyces* extracts (MUSC 93J, MUM 100J, MUM 147 J, MUM 176J, MUM 182 J, and MUM 197J) exhibited significant cytotoxic effect on Caco-2 cells with cell viability ranged from 74.8 ± 2.6% to 87.4 ± 5.3% (Fig. 7B). For SW480 cell line, 7 *Streptomyces* extracts (MUSC 1J, MUSC 93J, MUM 100J, MUM 147J, MUM 176J, MUM 182J, and MUM 197 J) demonstrated significant cytotoxicity against the cells with cell viability as low as 32.8 ± 7.7% to 87.4 ± 3.6% (Fig. 7C), with *Streptomyces* sp. MUM 197J exhibited up to 60–70% reduction of cell viability. It seems that most of the extracts tested had demonstrated significant cytotoxicity on HT-29 cells as illustrated in Fig. 7D. The 10 out of 18 *Streptomyces* extracts tested (MUSC 1J, MUM 91aJ, MUM 92J, MUSC 93J, MUM 100J, MUM 147J, MUM 180J, MUM 181J, MUM 182J, and MUM 197J) showed significant cytotoxic activity against HT-29 cells with cell viability ranged from 50.7 ± 0.9% to 89.4 ± 1.7%. *Streptomyces* sp. MUM 100J exhibited up to 40–50% reduction of cell viability.

## Discussion

A total of 88 *Streptomyces* isolates were obtained from soil samples collected at 7 sites in this study. Majority of these soil samples collected from the sites consisted of rhizosphere soils. Except for site KTTAS6, where sediment samples were also collected. Previous studies have shown that mangrove rhizosphere soils are rich in streptomycetes^77–80^. In the complex nature of mangrove rhizosphere environment, generation of root exudates could be the stimuli for species richness and chemical diversity of streptomycetes^78,79^. This rationalizes the higher number of *Streptomyces* isolates recovered from rhizosphere soil samples especially for those collected at sites KTTAS1, KTTAS2, and KTTAS3 as compared to that of sediment samples collected at site KTTAS6.

Findings of this study show that there is a high level of diversity within the genus *Streptomyces* present in mangrove of Sarawak. The isolates recovered from mangrove soil samples are related to various *Streptomyces* type strains, distributed into 7 major clades (Fig. 1). These type strains have been proven to produce useful bioactive compounds. For instances, *S*. *corchorusii* from marine sediment of Bay of Bengal in India (anticancer compounds)^61^ and *S*. *malachitofuscus* from soil of saline agricultural farmlands in Pakistan (antifungal compounds)^62^. This subsequently prompted the investigation of novel *Streptomyces* spp., resulting in the discovery of 18 potentially novel *Streptomyces* isolates (Fig. 2). Of the 18 putative novel isolates, MUSC 1J^T ^^25^ and MUSC 93J^T ^^26^ have been confirmed and reported as two distinct novel species through the application of polyphasic approach analyses. *Streptomyces monashensis* sp. nov. MUSC 1J^T^ was recognized by its significant antioxidative activity^25^; whilst *Streptomyces colonosanans* sp. nov. MUSC 93J^T^ was recognized by its colon healing properties^26^.

By referring to the phylogenetic relationships of the potentially novel *Streptomyces* isolates and their closely related type strains, further literature search was conducted to obtain additional information on the type strains. It was found that some of these type strains have been previously reported to produce useful bioactive metabolites. Hence, the 18 potentially novel *Streptomyces* isolates were selected for further investigation of their antioxidant and cytotoxic potentials through a customized high-throughput screening using a 96-well microplate.

Antioxidants are crucial for overcoming health issues caused by oxidative stress including cancer, diabetes mellitus, atherosclerosis, and neurodegenerative disorders^81–83^. Numerous techniques have been established for the examination of antioxidant activity of natural compounds due to the many different mechanisms involved in antioxidants^84^. The use of a single assay might be insufficient to determine the antioxidant ability, thus preliminary screening of the antioxidant activity of the extracts in this study was conducted using three different *in vitro* antioxidant assays: ABTS, metal chelating and SOD. These assays operate based on different principles, for which, ABTS measures the ability of scavenging free radicals, metal chelating estimates the ability to chelate metal ions, and SOD determines the inhibition of superoxide anion radical generation^85,86^ (https://www.sigmaaldrich.com/content/dam/sigma-aldrich/docs/Sigma/Datasheet/6/19160dat.pdf). Besides, ABTS, SOD and metal chelating assays are commonly used in many studies for the evaluation of antioxidant capacities of extracts. ABTS assay offers advantages such as rapid reaction with samples, additional flexibility in which it can work at varying pH levels as well as soluble in aqueous and organic solvents^87^. Meanwhile, metal chelating assay is conducted since chelation of metal ions is considered as one of the main mechanisms of antioxidant activity^88^. As for SOD assay, it is a simple experiment that can offer reproducible results^89^. Overall, the results of ABTS, SOD and metal chelating experiments demonstrated that the potentially novel *Streptomyces* isolates in this study generated significant antioxidant activity, and thus, the antioxidant traits of these isolates are worth to be further explored.

Phenolic compounds are recognized by their antioxidant activity and other bioactivities including anti-inflammatory, anti-microbial, and anti-allergenic^90–92^. As an initial attempt to determine whether phenolic compounds could be the contributors to the antioxidant activity produced by these streptomycetes, the TPC of *Streptomyces* methanolic extracts was evaluated to investigate the correlation of TPC and antioxidant activity. Based on the outcomes, it was unable to deduce that phenolic compounds were the major contributors to the antioxidant activity of these *Streptomyces* extracts due to the small correlation coefficient shown. Nonetheless, different samples of *Streptomyces* extracts were evaluated for the first time to establish the relationship between these two parameters. The results of this analysis could be limited by the small number of samples studied. Larger number of samples might be needed to further validate the relationship between these two parameters of the three antioxidant model systems. By increasing the sample size, there would be higher accuracy for the estimation of a correlation. In fact, the crude methanolic extracts may contain other compounds that are also accounted for their antioxidant activity. Perhaps in future studies, further experiments can be conducted for the determination of pure compound(s) responsible for the antioxidant activity exhibited by the methanolic extracts.

Globally, colorectal cancer ranked as the third most commonly diagnosed cancer^40,93,94^. Chemotherapy is one of the predominant methods to treat cancer, but there is limitation relating to the toxicities of the drugs used^49,95^. Hence, many ongoing efforts have been conducted by researchers around the world to search for effective chemotherapeutic agents in combating cancer. Human colon cancer cell lines with varied molecular characteristics (eg. HCT-116 cells contain wild type p53; HT-29, Caco-2, and SW480 contain mutated p53) were used as the panels in this study to examine the effectiveness of different extracts in inducing cytotoxicity against these cells^96–98^. The results of cytotoxic analysis revealed that majority of the *Streptomyces* extracts tested showed significant cytotoxicity against the colon cancer cells. The varying levels of cytotoxic activity observed could be due to the distinctive susceptibility or resistance of the colon cancer cell lines towards the extracts which contributed by their unique genetic makeup^26^.

In this study, rapid determination of bioactive isolates is established by the utilization of high-throughput screening systems which often involve performing various assays using 96-well plates^99,100^. One of the examples of such study was conducted by Hong *et al*.^99^ which involved five high-throughput screening models for the determination of antimicrobial, cell growth inhibition and enzymatic activities of over 2000 actinomycetes. The current findings unfolded many *Streptomyces* isolates exhibited at least one or both antioxidant and cytotoxic activities, thereby further supports that mangrove environments provide a rich source of bioactive streptomycetes.

Given the beneficial properties of streptomycetes can be offered towards mankind, it is encouraged that these microorganisms are introduced into genome programs in future studies. The advancement in next generation sequencing (NGS) technology offers high-throughput sequencing with more affordable price nowadays^101^. The genomic information obtained can be utilized for biosynthetic gene clusters mining for the determination of metabolite gene clusters capable of natural product biosynthesis, thus, exploiting the hidden potential of biosynthetic pathways^101–105^. Ultimately, the discovery of interesting compounds as potential drug leads is henceforth accomplishable.

## Conclusion

As a conclusion, the present study reveals the diversity of *Streptomyces* spp. from Sarawak mangrove based on a comprehensive genotypic and phylogenetic analyses. To our knowledge this is the first report on the diversity and bioactivities of streptomycetes from mangrove environment in Sarawak, East Malaysia. It is apparent that several of the *Streptomyces* isolates could merit novel species status, however, further investigation is required to confirm the novelty of the isolates. Other than the determination of possible novel species, the isolates demonstrated promising antioxidant and cytotoxic activities via a high-throughput bioactivity screening. This study has revealed untapped potential in Sarawak mangrove forest and it represents an invaluable source of streptomycetes that could be utilized for future bioprospecting studies. Additionally, the antioxidant and cytotoxic abilities presented by these mangrove-derived streptomycetes is a suggestive sign that they could be producing interesting bioactive compounds which may contribute to future drug discovery.

## Materials and Methods

### Environmental sampling

Collection of soil samples were conducted in June 2015 from mangrove forest at Kampung Trombol (Telaga Air) area of Kuching, state of Sarawak, Malaysia. Soil samples were obtained from 7 sites labelled as KTTAS 1 to KTTAS 7. At each site, three-sediment core samples were collected at a depth of approximately 20 cm (after removing 2–3 cm of surface soil) to make up one composite soil sample representing one site. The soil samples were placed into separate sterile 50 mL conical tubes using an aseptic metal trowel and stored in −20 °C freezer before transported to the laboratory^106^.

### Selective isolation and purification of *Streptomyces* spp

Soil samples were air-dried for about 7 days. Air-dried soil samples were thoroughly mixed using a mortar and pestle, after that selective pretreatment was conducted using wet heat at 50 °C for 15 minutes^107,108^. The pretreated soil samples were serially diluted (1:10 v/v) with sterilized water for up to 10^–4^. For each of the 10^−1^, 10^−2^, 10^−3^, and 10^−4^ soil suspensions, 100 µL of suspension was spread onto isolation media. Dilutions of suspensions were spread onto 11 different types of isolation media: ISP 2 (yeast malt agar), ISP 3 (oatmeal agar), ISP 4 (inorganic salt starch agar), ISP 5 (glycerol asparagine agar base), ISP 6 (peptone yeast extract 6 iron agar), ISP 7 (tyrosine agar base), *Streptomyces* agar (SA), starch casein agar (SCA), actinomycetes isolation agar (AIA), nutrient agar (NA), and Luria-Bertani agar (LB)^28,109–111^. All media were added with 50 mg/L of cycloheximide and 20 mg/L of nalidixic acid prior to experiment^26^. After the soil suspensions were spread onto the isolation media plates, the Petri dishes were incubated at 28 °C for 1–4 weeks. *Streptomyces*-like colonies with aerial mycelium and substrate mycelium features were selected and purified on ISP 2 medium. Maintenance of pure cultures included ISP 2 agar slants at 28 °C and glycerol suspensions (20%, v/v) at −20 °C^112^.

### Molecular identification of *Streptomyces* isolates

#### Genomic DNA extraction and PCR amplification of 16S rRNA gene

Genomic DNA extraction was performed according to the procedure adapted from Hong *et al*.^99^. PCR amplification of 16S rRNA gene was carried out in a final volume of 20 µL using TurboCycler 2 (Blue-Ray Biotech, Taipei, Taiwan) based on protocol adapted from Lee *et al*.^65^, with highQu Taq DNA polymerase (Kraichtal, Germany), universal primers P27F_BGI (5′-AGAGTTTGATCCTGGCTCA-3′) and P1492R_BGI (5′-GGTTACCTTGTTACGACTT-3′). The PCR cycling conditions were set as: initial denaturation at 95 °C for 5 minutes, 35 cycles of 94 °C for 50 seconds, 55 °C for 1 minute, 72 °C for 1 minute 30 seconds, and a final elongation at 72 °C for 8 minutes.

#### Phylogenetic analysis of 16S rRNA gene sequences

The 16S rRNA gene sequences obtained were manually trimmed using BioEdit Sequence Alignment Editor Software and aligned with representative sequences of closely related type strains in the genus *Streptomyces* obtained from GenBank/EMBL/DDBJ databases using CLUSTAL-X software^113^. The alignment was manually verified and adjusted before the reconstruction of phylogenetic tree. Phylogenetic tree was reconstructed with neighbour-joining algorithm using MEGA version 7.0 and the evolutionary distances for this algorithm were computed using Kimura’s two-parameter model^26,114^. The stabilities of the resultant tree topologies were analyzed through bootstrap analysis based on 1000 resampling method of Felsenstein (1985)^115^. Calculations of sequence similarity were performed by EzBioCloud server (http://www.ezbiocloud.net/)^116^.

### Fermentation and preparation of *Streptomyces* crude extracts

Each isolate was grown in tryptone soya broth (TSB) (BioMerge, Malaysia) as seed medium for 14 days prior to fermentation process. Fermentation was carried out in sterilized 200 mL Han’s Fermentation Media 1 (HFM1) (BioMerge, Malaysia) using a sterile 500 mL Erlenmeyer flask. The 14-day seed medium (200 µL) was inoculated into the fermentation medium and cultured at 28 °C, 200 rpm, for 10 days. Once fermentation was completed, the medium was subjected to centrifugation at 12000 g for 15 minutes, then the supernatant was filtered and collected. Freeze drying process was conducted on the clear filtrate, followed by extraction of freeze-dried sample using methanol for 72 hours and then re-extraction for twice at 24 hours intervals under same condition. The methanol containing extract was collected and concentrated by removing extracting solvent using a rotary vacuum evaporator at 40 °C. Final extract was suspended in dimethyl sulphoxide (DMSO) before proceeding to bioactivity screening^26,33^.

### Bioactivities screening of *Streptomyces* crude extracts

#### Antioxidant activity screening assays

The 2,2′-azino-bis (3-ethylbenzothiazoline-6-sulphonic acid) (ABTS) assay was performed to determine the antioxidant potential of the *Streptomyces* extract. The procedure for ABTS assay was previously described by Law *et al*.^26^. Briefly, ABTS radical cation (ABTS·) was produced by the reaction between ABTS stock solution (7 mM) and potassium persulphate (2.45 mM) for 24 hours. ABTS radical solution was added into the extracts (final concentration of 4 mg/mL in each well) preloaded in a 96-well microplate, with gallic acid as the positive control. The absorbance was measured at 743 nm using a microplate reader and the percentage of ABTS scavenging activity was calculated as follow^117^:

$${\rm{Percentage}}\,{\rm{of}}\,{\rm{ABTS}}\,{\rm{scavenging}}\,{\rm{activity}}\,( \% )=\frac{{\rm{Absorbance}}\,{\rm{of}}\,{\rm{control}}-{\rm{Absorbance}}\,{\rm{of}}\,{\rm{sample}}}{{\rm{Absorbance}}\,{\rm{of}}\,{\rm{control}}}\times 100 \% $$

Metal chelating activity of the *Streptomyces* extracts was investigated based on previously established method^26,117^. FeSO_4_ (2 mM) was added into extracts (final concentration of 4 mg/mL in each well) preloaded in a 96-well microplate and subsequently ferrozine (5 mM) was added to start the reaction. The positive control included was EDTA. Absorbance was measured at 562 nm using a microplate reader and percentage of metal chelating activity was calculated as follows^117^:

$${\rm{Percentage}}\,{\rm{of}}\,{\rm{metal}}\,{\rm{chelating}}\,{\rm{activity}}\,( \% )=\frac{{\rm{Absorbance}}\,{\rm{of}}\,{\rm{control}}-{\rm{Absorbance}}\,{\rm{of}}\,{\rm{sample}}}{{\rm{Absorbance}}\,{\rm{of}}\,{\rm{control}}}\times 100 \% $$

Superoxide dismutase/superoxide anion scavenging (SOD) activity of the extracts was examined according to established method with the use of a commercially available SOD assay Kit-WST (Sigma-Aldrich)^26,117^. The reaction solutions were added into extracts (final concentration of 2 mg/mL) preloaded in a 96-well microplate according to the manufacturer’s instructions. The absorbance was measured at 450 nm and the percentage of SOD activity was calculated as follows^26^:

$${\rm{Percentage}}\,{\rm{of}}\,{\rm{SOD}}\,{\rm{activity}}\,( \% )=\frac{(({\rm{Abs}}\,{\rm{control}}\,{\rm{blank}}\,1-{\rm{Abs}}\,{\rm{buffer}}\,{\rm{blank}}\,3)-({\rm{Abs}}\,{\rm{sample}}-{\rm{Abs}}\,{\rm{sample}}\,{\rm{blank}}\,2))}{({\rm{Abs}}\,{\rm{control}}\,{\rm{blank}}\,1-{\rm{Abs}}\,{\rm{buffer}}\,{\rm{blank}}\,3)}\times 100 \% $$

#### Determination of total phenolic content

The total phenolic content in the extracts was analyzed using the Folin-Ciocalteu’s reagent method according to the procedure described by Tan *et al*.^117^. Folin-Ciocalteu’s reagent was diluted 1:10 v/v with sterilized water prior to the experiment. The assay was conducted in a 96-well plate loaded with 10 µL of extracts in respective wells. Then, 50 µL of diluted Folin-Ciocalteu’s reagent was added into each well containing the extract and incubated in the dark for 5 minutes at room temperature. After incubation, 7.5% sodium carbonate (40 µL) was added into each well and incubated for 30 minutes at room temperature. Final absorbance was measured at 750 nm using a microplate reader and the data was expressed in gallic acid equivalents (GAEs).

### Maintenance and growth condition of human derived cancer cell lines

The human colon cancer cell lines obtained for the current study were maintained in RPMI (Roswell Park Memorial Institute)-1640 (Gibco, United States) supplemented with 10% fetal bovine serum and 1x antibiotic-antimycotic (Gibco, United States) in a humidified incubator at 37 °C with 5% CO_2_, 95% air^26^.

### Cytotoxic activity screening using 3-(4,5-dimethylthazol-2yl)-2,5-diphenyl tetrazolium-bromide (MTT) assay

The human colon cancer cell lines tested in this study were HT-29, Caco-2, HCT-116 and SW480. MTT assay was used for the investigation of cytotoxic activity the extracts according to protocol reported by Ser *et al*.^66^. Cells were seeded into a sterile 96-well microplate at a density of 5 × 10^3^ cells/well and allowed to adhere by overnight incubation at 37 °C in humidified atmosphere with 5% CO_2_, 95% air. Extract was added to the cells with final concentration 400 µg/mL and further incubated for 72 hours prior to MTT assay. DMSO at 0.05% (v/v) was included as negative control, while curcumin was included as positive control. MTT (5 mg/mL) (Sigma) was pipetted to each well and incubated for 4 hours. Then, the medium was gently aspirated and DMSO was added to dissolve the formazan crystals. The amount of formazan product was determined by measuring the absorbance at 570 nm (with 650 nm as reference wavelength) using a microplate reader. The percentage of cell viability was calculated according to the formula as shown below:

$${\rm{Percentage}}\,{\rm{of}}\,{\rm{cell}}\,\mathrm{viability}\,( \% )=\frac{{\rm{Absorbance}}\,{\rm{of}}\,{\rm{treated}}\,{\rm{cells}}}{{\rm{Absorbance}}\,{\rm{of}}\,{\rm{untreated}}\,{\rm{cells}}}\times 100 \% $$

### Statistical analysis

Antioxidant and cytotoxic screening assays in this study were conducted in quadruplicate. The data was stated as mean ± standard deviation (SD). One-way analysis of variance (ANOVA) with subsequent Tukey’s post hoc analysis was conducted using SPSS statistical analysis software version 22 to verify the significant differences between groups. A difference was considered statistically significant when *p* ≤ 0.05.
